# Tool for identifying occupational exposures and risks in agriculture (TIERRA): application in coffee farming in Uganda

**DOI:** 10.3389/ijph.2026.1609449

**Published:** 2026-06-04

**Authors:** Hannah Wey, Lena Jäggi, Aggrey Atuhaire, Felix Boos, Peter Ssekkadde, Sandra Abeine, Ruth Mubeezi, Samuel Fuhrimann

**Affiliations:** 1 Swiss Tropical and Public Health Institute (Swiss TPH), Basel, Switzerland; 2 Universitat Basel, Basel, Switzerland; 3 Uganda National Association of Community and Occupational Health (UNACOH), Kampala, Uganda; 4 ETH-Bereich Hochschulen, Zürich, Switzerland; 5 Independent Researcher, Kampala, Uganda; 6 Makerere University College of Health Sciences, Kampala, Uganda

**Keywords:** Delphi, occupational hazards, prioritization, smallholder farmers, stakeholder engagement

## Abstract

**Objectives:**

This study developed a practical, context-sensitive assessment tool to prioritize occupational risks in agriculture (TIERRA). We applied TIERRA in a case study with smallholder coffee farmers in Uganda (FarmCoUganda) to demonstrate its effectiveness.

**Methods:**

TIERRA follows a stepwise, participatory process beginning with an inventory of 80 occupational hazards, reviewed by local experts for clarity and prioritized by community stakeholders through a Delphi workshop. Participants rate the likelihood, severity, and risk of hazards. In the Ugandan case study, 31 stakeholders rated 59 hazards relevant to smallholder coffee farmers in Mbale District.

**Results:**

Stakeholders prioritized 23 hazards across six categories: biological, chemical, ergonomic, and physical hazards, each comprising three items, plus five environmental and six psychosocial hazards. “Contact with a pesticide” received the highest overall risk score. “Mosquito bites” emerged as the most significant biological risk. Ergonomic hazards received the highest categorical risk rating.

**Conclusion:**

TIERRA fosters stakeholder ownership and facilitates dialogue toward action. It supports the development of tailored farmer surveys, training and safer working conditions. It offers a methodological blueprint for adaptation across diverse agricultural contexts, including in LMICs.

## Introduction

Agriculture plays a crucial role in economic development, providing employment for approximately 874 million people globally [1]. As such, it contributes directly to Sustainable Development Goals (SDGs) 1 (“No Poverty”) and 8 (“Decent Work and Economic Growth”). However, the sector’s contributions to development come with significant sustainability and human health trade-offs. The agricultural sector is one of the most hazardous industries worldwide [2], with farmers being exposed to numerous health and safety hazards, including biological, chemical, ergonomic, physical, environmental, and psychosocial challenges [3–5]. These occupational hazards are particularly acute in low- and middle-income countries (LMICs), where the majority of agricultural labor is informal and underregulated.

Improving occupational health in agriculture is not only a matter of individual wellbeing but also central to building more sustainable and resilient food systems. Training programs—such as farmer field schools, Non-Governmental Organizations (NGOs) initiatives, and certification schemes—are often deployed to reduce occupational risks by improving farmers’ knowledge and promoting safer practices, for example, in pesticide use [6–8]. However, the effectiveness of such training depends on how well it addresses the actual hazards farmers face within specific production systems and socio-economic contexts. Although there is broad consensus that agriculture involves multiple and interacting occupational hazards, evidence remains limited on how to systematically identify and prioritize the most critical hazards in a given production context, particularly in smallholder settings in LMICs [9]. Consequently, it is often unclear whether existing trainings are sufficiently tailored to local production realities, address the most important occupational hazards, and effectively improve farmers’ physical, mental, and economic wellbeing [8, 10].

One key barrier to improving risk mitigation is the lack of a practical, context-sensitive assessment tool that informs evidence-based interventions. While broad frameworks exist—for example those developed by the International Labour Organization (ILO) [1, 2, 4, 11]—they are rarely tailored for application in diverse smallholder settings and often lack the specificity needed for operational decision-making. As a result, they may not adequately capture the hazard profiles of particular agricultural systems or help determine which risks should be prioritized in a given context. There is therefore a need for a simple, standardized, yet flexible tool that can be adapted to different production environments and used with stakeholders to identify and prioritize the most relevant occupational hazards.

This study addresses that need by describing the development of such a tool and its adaptation to a specific agricultural context. We present a practical and transferable approach for quantifying and prioritizing occupational hazards with stakeholder input, and we apply it in a case study of smallholder coffee-farming in Uganda. In doing so, we aim to demonstrate the tool’s usefulness for identifying context-relevant occupational risks and informing more targeted research and interventions to improve farmers’ health and safety.

Uganda is among the world’s leading coffee producers [12], with green (unroasted) coffee accounting for 14.5% of the country’s total export value between 2014 and 2023 [13]. In the 2023/24 fiscal year, revenue from Ugandan coffee exports rose to USD 1.1 billion [14–16]. In addition, coffee production is a critical livelihood source for an estimated 12 million Ugandans, the majority of whom are smallholder farmers [17].

Despite its economic importance, smallholder coffee production is associated with a high burden of occupational hazards [18]. These are exacerbated by systemic vulnerabilities such as limited access to health services and information, low levels of education, poor infrastructure, and limited financial resources. In Uganda, many smallholder farmers live below the income threshold for extreme poverty, facing conditions that barely ensure their survival [17]. Finally, external stressors, such as volatile coffee prices, climatic stressors and increased incidence of pests and diseases, further compound the challenges faced by smallholder coffee farmers [19].

Beyond this case study, our methodological blueprint can support researchers in adapting the tool to other agricultural contexts, with the broader aim of improving occupational health, reducing injury, and fostering safer, more sustainable rural livelihoods [4].

## Methods

### Study area

Mbale is a Ugandan district situated on the volcanic slopes of Mount Elgon. The high altitude, fertile volcanic soil, and a humid tropical climate provide ideal conditions for the cultivation of Arabica coffee, which can fetch higher export prices compared to Robusta varieties due to its higher quality [20, 21]. Smallholder farming communities predominantly populate the region in scattered homesteads, managing mixed agroforestry systems on plots typically smaller than 1 ha [22, 23]. For these farming families, selling green coffee is often their primary source of cash income. However, with limited resources to buffer against fluctuations in harvests, many smallholder farmers across Uganda and in the Mount Elgon area are in an economically precarious situation [19].

### Tool development (TIERRA)

In a first step we carried out a bibliographic review to identify all occupational hazards in agriculture and specifically in coffee [2–4, 11, 18, 24–34] followed by a rapid assessment in the field, resulting in a list of 80 possible hazards items.

**FIGURE 1:**
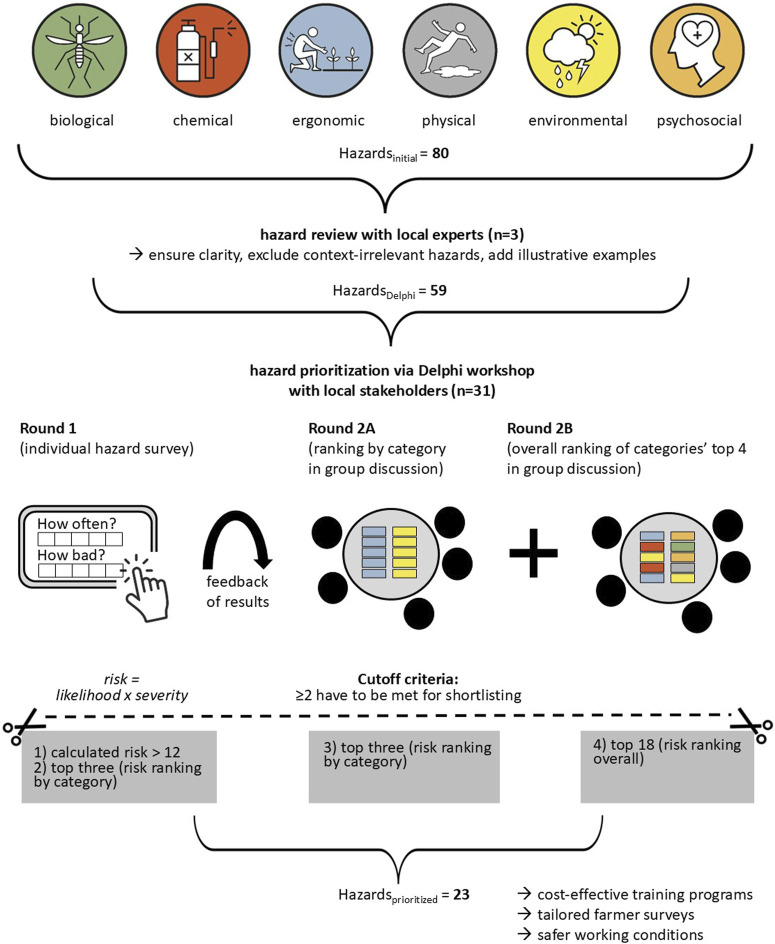
Stepwise process of the “Tool for Identifying occupational ExposuRes and Risks in Agriculture” (TIERRA) (Mbale, Uganda. 2024).

The hazard questionnaire was then pre-tested with three local experts (Figure 1). Modifications based on that local feedback included the exclusion of hazards deemed not applicable to the local context, such as those associated with the use of large tools and machinery (e.g., gas bottles, vibrating tools) which are not commonly used in the region, as well as hazards unrelated to the type of work, such as “shift work” and “long commuting distances”. Several hazards were merged to improve clarity, for example, on weather perception (e.g., cold and rainy weather, sunshine and heat). In addition, local examples were added for specific hazards to enhance understanding and illustrate the hazard, e.g., “with knife, panga machete, hand-hoe, nails” for the physical hazard “accident with a sharp tool”. This resulted in a final list comprising 59 hazard items, (H_overall_ = 59) grouped into the six categories “biological” (h_bio_ = 10), “chemical”, (h_chem_ = 8), “ergonomic” (h_ergo_ = 5), “physical” (h_phys_ = 8), “environmental” (h_env_ = 8), and psychosocial (h_psycho_ = 20) (Figure 1).

### Workshop procedure

The tool was applied as part of a farmer cohort study in the Mbale region (FarmCoUganda). Following the compilation of the occupational hazard list, we conducted a one-day in-person workshop with local stakeholders using a modified two-stage Delphi method. The goal was to prioritize and refine the long list of occupational hazards into a locally grounded set of the most relevant risks for coffee farmers in the Mbale district.

#### Rationale for a modified two-stage Delphi method

The Delphi method is a structured group communication technique designed to generate consensus among a panel of experts through iterative rounds of feedback and reflection [35–37]. Delphi methods can be used in any area in which expertise is needed to come to consensus around a particular issue [38, 39]. Traditional Delphi studies are often conducted remotely over multiple rounds, using quantitative survey data to refine opinion and build consensus over time. Given the local realities in our setting, including limited digital access, potential difficulties interpreting quantitative summary statistics, and a strong cultural preference for in-person engagement, we adapted the approach to be conducted within a one-day, face-to-face workshop. This design enabled direct interaction, clarification of concepts, and facilitated real-time discussion. Unlike traditional Delphi studies that prioritize consensus-building, our approach emphasized participatory engagement, building trust, and fostering a sense of ownership and involvement in the project.

#### Participant selection

Participants were selected to reflect a broad range of expertise related to smallholder coffee farming in Mbale district to capture diverse perspectives. Local stakeholders were identified by the project coordinator in consultation with the Chief Administrative Officer of Mbale District. To be eligible, participants needed to have at least 2 years of experience working directly with coffee producers in the region. The 34 invited stakeholders included relevant local government technical leaders, representatives from farmer unions and cooperatives, local research institutions, the private sector and NGOs. They were invited at least 3 weeks prior to the workshop through official hand-delivered letters and followed up through phone call reminders. The workshop was held on 31 July 2024 at a central venue in Mbale, Uganda.

This expert-based approach was the first step in a multi-stage process, in which stakeholders were engaged to prioritize hazards before subsequent data collection with farmers. While not reported in this paper, a second step (a field-based assessment of hazard exposure, as self-reported by farmers through a questionnaire survey) was conducted following the workshop. This sequential approach improves feasibility by reducing respondent burden and enhances stakeholder ownership and the co-creation of locally relevant knowledge.

#### Delphi rounds and procedures

The workshop followed a Delphi approach consisting of two stages (Round 1 and Rounds 2A/2B in Figure 1).

#### Round 1: individual hazard rating

Participants completed a structured hazard questionnaire individually, either on their mobile phones or on tablets provided by the research team. Each participant rated a list of occupational hazards in coffee farming along two dimensions:

- Likelihood: “What is the likelihood of a coffee farmer encountering this hazard during work on the farm in the last 12 months?”
- Severity: “If it occurs, how bad is it for the farmer’s health, on average?”

Both dimensions were assessed on five-point Likert scales (1 = rare/no harm, 5 = almost certain/potentially fatal). Participants were instructed to consider typical, rather than extreme, outcomes and to factor in both short- and long-term health effects. This round served as the initial, independent input of expert opinion and formed the basis for group-level discussions in Round 2.

#### Feedback presentation

Following Round 1, the aggregated results (mean values of likelihood and severity for each hazard) were presented to all participants (Figure 1). This feedback provided a shared evidence base for subsequent discussion and allowed participants to compare their own assessments with group-level trends.

#### Round 2A: risk ranking by category in groups

Following the individual assessment of likelihood and severity per hazards in Round 1, participants were divided into six breakout groups, each composed of 5–6 individuals with intentionally mixed professional backgrounds. Facilitated by the workshop leader, each group received printed labels listing the hazards by pre-established categories (biological, chemical, ergonomic, physical, environmental, psychosocial). To support cognitive processing, each hazard category was color-coded (e.g., green for biological hazards). Groups discussed and ranked the hazards within each category based on perceived overall risk, combining likelihood and severity. This phase encouraged participants to reflect on their individual assessments, share and integrate domain-specific expertise, and discuss any disagreements or ambiguities.

#### Round 2B: cross-category prioritization in groups

In the final phase, each group selected the top four most relevant hazards per category identified in their Round 2A and ranked them according to their overall relevance across all categories (Figure 1).

### Cutoff criteria for hazard prioritization

To develop a concise and relevant list of occupational hazards for use in a future cohort study, we applied a structured approach to prioritization. The goal was to reduce the number of hazards to a manageable set (no more than 25) while retaining those most relevant to the local farming context. This reduction was necessary to minimize the burden on study participants, many of whom have limited experience with surveys and low education level, and to focus the assessment on hazards that are both significant and recognizable.

To determine which hazards to retain, we applied four predefined criteria. A hazard was shortlisted if it met at least two of the following (Figure 1):

1. Mean risk score in Round 1 exceeded 12.
2. Ranked among the top three hazards by risk score within its category in Round 1.
3. Ranked among the top three hazards within its category in Round 2A group discussions.
4. Included among the top 18 overall priorities in Round 2B cross-category rankings.

For Round 2A, average rankings within each category were calculated across the six discussion groups. In Round 2B, hazards were selected based on group consensus, and the number of groups selecting each hazard, along with the average rank across those groups, was recorded. Hazards not selected in Round 2B were not assigned a ranking.

This combined analytic and prioritization process ensured that the final list of hazards reflected both individual assessments and group-level consensus, balancing statistical rigor with practical relevance for smallholder coffee farmers in the region.

### Statistical analysis

All Round 1 questionnaire responses were collected digitally using Open Data Kit (ODK) and analyzed in RStudio (version 2024.09.0). Descriptive statistics (counts, means, and standard deviations) were used to summarize participant characteristics. For each hazard, a risk score was calculated per participant by multiplying the likelihood and severity ratings. Mean and standard deviation values for likelihood, severity, and risk were then computed for each hazard and across all participants.

## Results

### Participant characteristics

Out of 34 stakeholders contacted, 31 participated in the workshop (91%). The group included agricultural extension officers, public and occupational health professionals, representatives from the coffee sector and NGOs, as well as smallholder farmer leaders. The mean age was 44 years, and 42% were female (Table 1).

**TABLE 1:** Characteristics of the local stakeholders participating in the Delphi workshop (Mbale, Uganda. 2024).

Participating stakeholders	-	-	31 (100%)
Age	Mean (standard deviation)	-	44.1 (9.3)
Age group	Counts	18–30 years	4 (12.9%)
​	​	30–50 years	15 (48.4%)
​	​	≥50 years	12 (38.7%)
Gender	Counts	Female	13 (41.9%)
​	​	Male	18 (58.1%)
Education	Counts	Primary	1 (3.2%)
​	​	Ordinary	4 (12.9%)
​	​	Advanced	2 (6.5%)
​	​	Bachelor	17 (54.8%)
​	​	Master	6 (19.4%)
​	​	PhD	1 (3.2%)
Occupation	Counts	Government	18 (58.1%)
​	​	Knowledge broker (e.g., university)	1 (3.2%)
​	​	Private sector	5 (16.1%)
​	​	Advocacy actor (e.g., NGOs)	3 (9.7%)
​	​	Self-employed	4 (12.9%)
Coffee grower	Counts	Yes	19 (61.3%)
​	​	No	10 (32.3%)
​	​	Not any more	2 (6.5%)
Self-estimated knowledge Evaluated after round 1 using a likert scale from 1 (low) to 5 (high)	Mean (standard deviation)	Biological	3.9 (0.9)
	​	Chemical	3.6 (1.3)
	​	Ergonomic	3.5 (1.0)
	​	Physical	4.0 (0.8)
	​	Environmental	3.8 (1.1)
	​	Psychosocial	3.5 (1.1)
	​	Overall knowledge confidence	3.9 (0.7)

### Individual assessment of likelihood and severity in the hazard questionnaire tool (Round 1)

The likelihood of occupational hazards, given as means on a Likert scale from 1 (rare) to 5 (almost certain) and standard deviations, ranged from 2.4 ± 1.2 (“exposure to forest fire”/“attack by a wild animal”) to 4.3 ± 0.98 (“prolonged bending”). Severity scores, also given as means on a Likert scale from 1 (no harm) to 5 (potentially fatal) with standard deviations, varied from 2.4 ± 1.1 (“contact with a disinfectant”) to 4.3 ± 0.9 (“contact with exposed electrical connections”).

**FIGURE 2:**
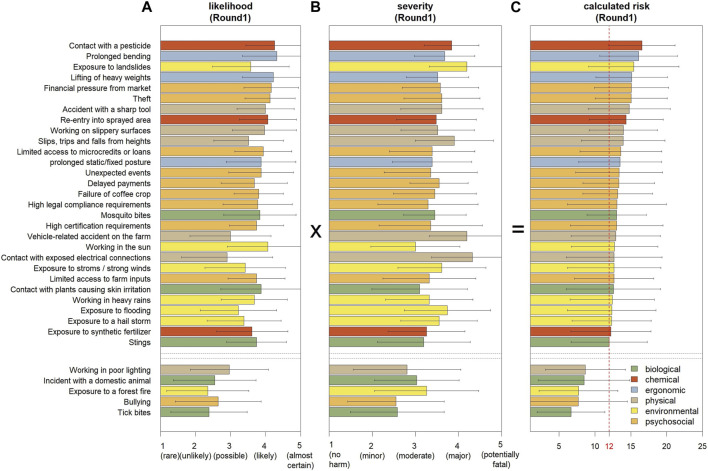
Likelihood **(A)**, severity **(B)**, and calculated risk as a product of these two assessments **(C)** for the top 29 and bottom 5 hazards, shown above and below the double dotted line, respectively, out of an initial set of 59 hazard items. Likelihood and severity were rated on Likert scales from 1 (low) to 5 (high). Results are presented as means with standard deviations. Bar colors indicate hazard categories. In panel **(C)**, the red dotted line marks the Round 1 threshold for shortlisting hazards (criterion 1), defined as a calculated risk value greater than 12 (Mbale, Uganda. 2024).

Calculated risk values, integrating likelihood and severity, spanned from 6.7 ± 4.7 (“tick bites”) to 16.5 ± 4.6 (“contact with a pesticide” from the chemical category) (Figure 2, Supplementary Material 1). The hazards presented in the upper part of Figure 2 were shortlisted as they met the cutoff criteria for Round 1, i.e., had a calculated risk value exceeding 12, and ranked among the top three per category based on risk values. The second criterion added hazards in the psychosocial and environmental category. With 13.0 ± 4.1, the “mosquito bites” showed the lowest standard deviation of all hazards, while “high legal compliance requirements” showed the highest standard deviation (13.1 ± 6.9).

### Categorical hazard ranking based on group discussion (Round 2A)

**FIGURE 3:**
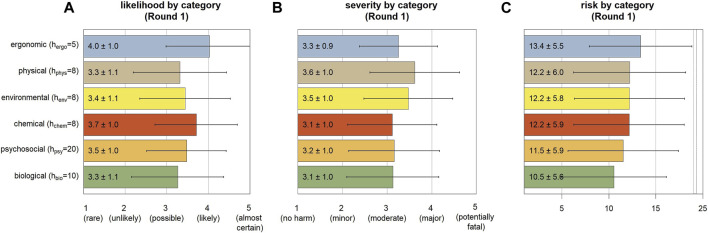
Categorical means and standard deviations for the likelihood **(A)**, severity **(B)** and calculated risk **(C)** for Round 1 (Mbale, Uganda. 2024).

During Round 2A the environmental and psychosocial categories were subject to extensive debate, leading to considerable rearrangement of hazard priorities compared to the individual ranking in Round 1 (Figure 3). As a result, and contrary to the ranking in Round 1 (criterion 2), the hazards “working in heavy rains” and “working in a cold environment” from the environmental category were prioritized in Round 2A. These additional hazards had also already met criterion 1 (risk >12 in Round 1). The same applies to “unexpected events”, “failure of coffee crop” and “limited access to farm inputs” in the psychosocial category (Figure 3).

**FIGURE 4:**
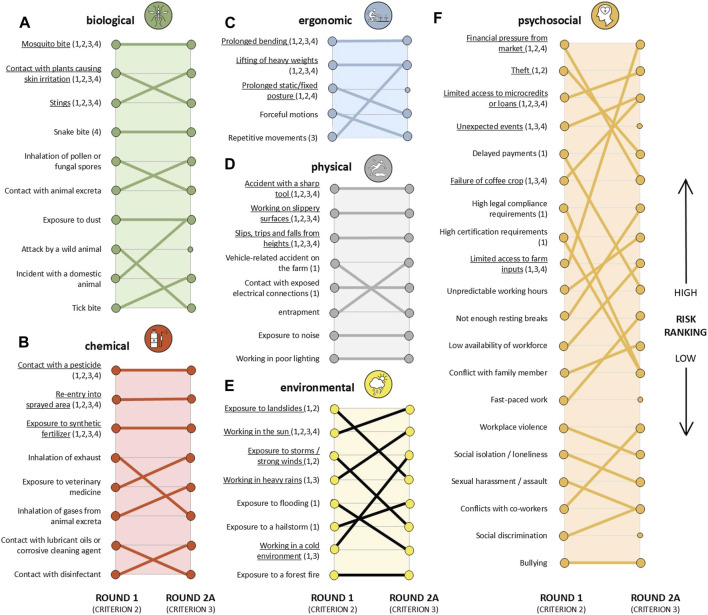
Comparison of categorical risk rankings between Round 1 (Criterion 2) and Round 2A (Criterion 3) in the categories of biological **(A)**, chemical **(B)**, ergonomic **(C)**, physical **(D)**, environmental **(E)**, and psychosocial **(F)** hazards. Both criteria assess whether a risk ranked among the top three in each category within the respective round. In Round 1, the ranking was based on the calculated risk from individual survey responses, where participants rated the likelihood and severity of each hazard. In Round 2A, rankings were developed through group discussions at each table. The values shown represent the average ranking across all tables. Hazards are annotated with the criteria they meet (in brackets). The hazards that were finally shortlisted meeting at least two of the four criteria are underlined (Mbale, Uganda. 2024).

In contrast, there was broad agreement in the other categories (biological, chemical, ergonomic, physical), and the top three hazards remained largely unchanged. “Repetitive movements” in the ergonomic category fulfilled criterion 3, but not shortlisted as it did not fulfil the other criteria (Figure 4).

### Overall hazard ranking based on group discussion (Round 2B)

Results from Round 2B (criteria 4) confirmed the prioritization established in Round 1 and Round 2A. Applying the initial rule that at least two cutoff criteria had to be met for a hazard to be shortlisted, a total of 23 hazards were selected (Figure 4).

## Discussion

This study aimed to describe the development of a practical, context-sensitive tool for identifying and prioritizing occupational hazards in agriculture, and to demonstrate its application in the context of smallholder coffee farming in Uganda. Specifically, using a Delphi approach during an in-person workshop with 31 local stakeholders, we showed that this process can be used to prioritize a broad set of occupational hazards, narrowing an initial list of 59 hazards to 23 priority hazards across six categories.

### Hazard prioritization

Stakeholders prioritized hazards across six categories, selecting three biological, three chemical, three ergonomic, and three physical hazards, as well as five environmental and six psychosocial hazards. The hazard “contact with a pesticide”, e.g., during mixing, loading, transport, storage, application, or disposal, was consistently ranked as the top hazard across all assessment rounds (Figures 2, 4, Supplementary Material 1). The related hazard, “re-entry into sprayed areas,” was also ranked highly. This is consistent with broader concerns about pesticide exposure in LMICs: in Uganda’s tropical climate, coffee growing is affected by numerous biotic constraints, including insect pests, diseases, weeds, and nematodes [40, 41]. Farmers therefore rely on synthetic pesticides as their primary, and sometimes only, means of crop pest and disease control. However, recommended safety precautions are often not followed [6, 7, 42–47]. In addition, some of the pesticide products used may contain active ingredients that are internationally banned, although evidence for this in Uganda is mixed, compared with more consistent findings reported from other developing countries [45, 47–49].

The extent of pesticide use among Ugandan smallholder farmers, however, varies considerably across studies and regions. Fuhrimann et al., for example, reported that 72% of smallholder farmers in a study in Wakiso district in Central Uganda used pesticide products that posed substantial health risks [50]. By contrast, Kagezi et al. concluded that pesticide use was generally limited in Mount Elgon region, with only 19% of coffee farmers using pesticides [49]. The contrasting findings and the high likelihood score assigned by stakeholders in our workshop (4.3 ± 0.8, corresponding to “likely to almost certain”) suggests either substantial regional variation in pesticide use or a possible overestimation of regional pesticide spraying for Arabica coffee by workshop participants possibly influenced by their experience with other crops and regions. Given the mixed farming systems common in the region, it also remains unclear to what extent pesticide use on non-coffee crops may have shaped the expert assessment.

Information on the specific pesticide active ingredients used in Ugandan smallholder systems remains limited and fragmented. Available studies indicate the use of the herbicide glyphosate, the fungicide mancozeb, and the insecticides cypermethrin [45, 51, 52] and fenitrothion [53]. More recently, Uganda’s Ministry of Agriculture, Animal Industry and Fisheries (MAAIF) has severely restricted the use of the insecticides imidacloprid and thiamethoxam on coffee, reflecting safety concerns [54]. Glyphosate and cypermethrin, in particular, have been associated with measurable internal exposure among farmers in urinary biomarker studies, and have been linked to adverse health outcomes, including sleep disturbances [51, 52]. Most pesticide active ingredients used by Ugandan farmers fall into WHO hazard class II, indicating moderate toxicity [45].

“Exposure to landslides” (15.4 ± 6.3) stood out in Round 1 due to its particularly high severity score (4.2 ± 0.9) (Figure 2). This is consistent with the Mount Elgon region being one of the most landslide-prone areas in Africa, where landslides contribute to fatalities, casualties, land loss, soil degradation, damage to property and infrastructure, and reduced agricultural productivity [55–57].

“Mosquito bites” was rated as the highest biological risk (13.0 ± 4.1), likely reflecting both farmers’ frequent outdoor exposure to mosquitoes, and the country’s high malaria burden. The relatively low standard deviation suggests strong agreement among workshop participants, possibly because malaria and its health consequences are widely recognized through public health campaigns like the Uganda National Malaria Control Program (UNMCP) [58].

Ergonomic hazards received the highest ratings among all hazard categories in Round 1 (13.4 ± 5.5 in Figure 3). This is not surprising, given that agricultural workers in LMICs frequently experience musculoskeletal disorders linked to physically demanding occupational tasks [59]. In coffee production, these risks are particularly prevalent during harvest, when workers perform repetitive movements in awkward postures and carry heavy bags of coffee berries, contributing to pain symptoms [59–63]. The literature also highlights wider consequences of these issues, including absenteeism, increased production costs, and long-term reductions in productivity [64, 65]. However, assistive equipment for coffee harvesting is currently limited. One promising solution is an ergonomic redesign of the collection bag [60, 66]. Mechanized harvesting alternatives, such as vibrating machines used for other crops like cherries and olives, could further reduce the ergonomic strain. However, these approaches face substantial technological and financial challenges. Key obstacles include the difficulty of distinguishing ripe from unripe fruit [67], the operation of heavy equipment on steep and uneven terrain without causing soil damage, dependence on fuel or electricity [68], and prohibitively high initial costs.

Psychosocial factors such as stress, fatigue, and long working hours may further contribute to pain symptoms alongside mechanical strain [59, 69]. The workshop discussions around health impacts in the psychosocial category (Figure 3), might suggest that there is a lack of awareness and understanding around causes and indirect health consequences of stress, and that these risks therefore remain insufficiently understood.

### Strengths & limitations

A strength of this study is the participation of 31 stakeholders in the workshop, which constitutes an adequate panel size [37, 39]. Another notable strength is the integration of gender perspectives in a context where coffee production is typically male dominated. Previous studies conducted with coffee farmers on the northern slopes of Mount Elgon have reported a share of female coffee farmers ranging from 10% to 47% [22, 49, 70]. In contrast, female stakeholders comprised 42% of participants in our workshop. However, among these female participants, direct experience with practical coffee cultivation was notably lower than that of their male counterparts (Table 1).

On the other hand, there may be several limitations in the assessment related to how the hazards and their consequences are interpreted by the stakeholders in the workshop. These limitations arise from differing conceptual understandings in four key areas: (1) single events versus repeated exposure, (2) permanent versus reversible health effects, (3) short-term versus long-term health impacts, and (4) averaging all possible health impacts on a Likert scale. We attempted to address the first point and improve the accuracy of likelihood ratings by carefully introducing the topic and providing illustrative examples for each hazard. However, severity assessments may still be influenced by how stakeholders perceive the reversibility and duration of effects and how they mentally average these impacts. The same applies to the overall risk concept applied in Round 2, which encompasses both likelihood and severity.

To further verify the assessment, we need to ask farmers directly about their exposures, for example, regarding pesticide use intensity and products, in the subsequent cohort study, both for Arabica coffee and other crops.

### Conclusion

We developed TIERRA, a practical and context-sensitive tool designed for prioritizing occupational risks in agriculture. This tool was successfully applied in the context of smallholder coffee farming in Uganda (FarmCoUganda), demonstrating its relevance in a real-world, resource-constrained setting.

Our participatory approach offers several advantages for occupational risk assessment in agriculture. It fosters local stakeholder ownership, supports the co-creation of knowledge, and encourages dialogue for action. Importantly, it ensures that the identified priorities are based on the lived experiences of farmers and reflect specific local conditions. Additionally, TIERRA allows for the development of tailored farmer surveys that focus only on relevant hazards, thereby reducing the burden on respondents. This makes data collection among farmers more focused and feasible in terms of time and logistics. Ultimately, improving the identification of critical occupational risks lays the foundation for evidence-based interventions and training programs that align with local realities and contribute to safer, healthier, and more sustainable agricultural livelihoods.

Our approach provides a methodological blueprint that can be easily adapted to other agricultural contexts, including resource-constrained settings in LMICs. The structured prioritization criteria offer a consistent framework for cross-context comparison, supporting broader efforts toward cleaner and more sustainable agricultural production.

## Data Availability

The data supporting the findings of this study are not openly available due to its sensitive nature and are available from the corresponding author upon reasonable request and under a data use agreement only. Data are located in controlled-access data storage at the Swiss Tropical and Public Health Institute.
