# Supplementary material for: Tool for identifying occupational exposures and risks in agriculture (TIERRA): application in coffee farming in Uganda
Source: Int J Public Health. 2026 Jun 4;71:1609449. doi: 10.3389/ijph.2026.1609449 (PMC13329147; doi:10.3389/ijph.2026.1609449)
Supplement: Supplementary file 1 [file Table1.docx]

## Supplementary information

Supplementary File 1: Result values per hazard. The sorting is according to how the hazards were presented to the Delphi participants in Round 1. Highlighted values indicate that the criterion is fulfilled. Hazards with *** annotated means these are shortlisted because they fulfill at least two preset criteria. A higher value, including the rankings, always indicates higher risk. For example, in "categorical risk ranking score" in Round 1, "10" is the highest ranked hazard according to the risk calculation, and "1" the lowest and last one.

|  |  | **Round 1  (H_overall_=59)** |  |  |  | **Round 2A** | **Round 2B** |  |
| --- | --- | --- | --- | --- | --- | --- | --- | --- |
|  |  | **likelihood  mean (sd)** | **severity  mean (sd)** | **(1) risk  mean (sd)** | **(2) categorical  risk ranking score** | **(3) mean categorical  risk ranking score** | **(4) mean categorical  risk ranking score** | **number of  groups** |
|  |  | 1 = rare 5 = almost certain | 1 = no harm 5 = potentially fatal | 1=lowest 25=highest | 1 = lowest h_category_ = highest | 1 = lowest h_category_ = highest | 1 = lowest 24 = highest | 0 = no group 6 = all groups |
| biological (h_bio_ = 10) | Snake bites | 3.0 (0.9) | 3.8 (0.9) | 11.5 (4.9) | 7 | 7.0 | 12.0 | 4 |
|  | Tick bites | 2.4 (1.1) | 2.6 (1.1) | 6.7 (4.7) | 1 | 2.2 | NA | 0 |
|  | *** Mosquito bites | 3.8 (1.0) | 3.5 (0.7) | 13.0 (4.1) | 10 | 9.3 | 13.4 | 6 |
|  | *** Stings | 3.7 (0.9) | 3.2 (1.1) | 12.0 (5.3) | 8 | 8.3 | 10.0 | 5 |
|  | Attack by a wild animal | 2.4 (1.2) | 3.5 (1.1) | 8.7 (6.3) | 3 | 1.5 | NA | 0 |
|  | Incident with a domestic animal | 2.5 (1.2) | 3.0 (1.0) | 8.5 (6.4) | 2 | 3.3 | NA | 0 |
|  | Contact with animal excreta | 3.8 (1.1) | 2.8 (1.1) | 10.7 (5.7) | 5 | 6.5 | 8.3 | 3 |
|  | Exposure to dust | 3.4 (1.4) | 2.9 (1.1) | 10.5 (6.5) | 4 | 3.3 | NA | 0 |
|  | Inhalation of pollen or fungal spores | 3.8 (1.1) | 3.0 (1.1) | 11.4 (5.3) | 6 | 6.3 | 7.3 | 2 |
|  | *** Contact with plants causing skin irritation | 3.9 (1.1) | 3.1 (1.1) | 12.6 (6.6) | 9 | 7.2 | 10.2 | 4 |
| chemical (h_chem_ = 8) | *** Contact with a pesticide | 4.3 (0.8) | 3.8 (0.6) | 16.5 (4.6) | 8 | 8.0 | 19.1 | 6 |
|  | *** Re-entry into sprayed area | 4.1 (0.8) | 3.5 (0.9) | 14.4 (5.2) | 7 | 6.3 | 15.6 | 6 |
|  | *** Exposure to synthetic fertiliser | 3.6 (1.0) | 3.3 (0.9) | 12.2 (5.6) | 6 | 6.2 | 13.0 | 6 |
|  | Exposure to veterinary medicine | 3.6 (0.9) | 3.1 (1.2) | 11.8 (6.2) | 4 | 5.2 | 7.4 | 4 |
|  | Inhalation of exhaust | 3.6 (1.1) | 3.1 (1.1) | 11.8 (6.5) | 5 | 2.5 | NA | 0 |
|  | Inhalation of gases from animal excreta | 3.8 (1.1) | 2.8 (1.2) | 11.4 (6.6) | 3 | 4.2 | 6.3 | 2 |
|  | Contact with lubricant oils or corrosive cleaning agents | 3.4 (1.0) | 2.9 (0.9) | 10.5 (5.6) | 2 | 1.7 | NA | 0 |
|  | Contact with desinfectant | 3.3 (1.2) | 2.4 (1.1) | 8.8 (6.7) | 1 | 2.0 | NA | 0 |
| ergonomic (h_erg_ = 5) | *** Lifting of heavy weights | 4.2 (0.9) | 3.5 (0.7) | 15.1 (5.0) | 4 | 3.2 | 10.9 | 6 |
|  | *** Prolonged bending | 4.3 (1.0) | 3.7 (0.7) | 16.1 (5.4) | 5 | 5.0 | 16.9 | 6 |
|  | *** Prolonged static/fixed posture | 3.9 (1.0) | 3.4 (0.9) | 13.5 (5.8) | 3 | 2.0 | 10.0 | 2 |
|  | repetitive movements | 3.9 (1.3) | 2.8 (1.0) | 11.0 (5.6) | 1 | 3.2 | 8.7 | 6 |
|  | Forceful motions | 3.8 (1.0) | 2.9 (1.0) | 11.2 (5.5) | 2 | 1.7 | 7.4 | 4 |
| physical (h_phy_ = 8) | *** Accident with a sharp tool | 4.0 (0.8) | 3.6 (1.0) | 14.8 (5.7) | 8 | 7.7 | 18.4 | 6 |
|  | Entrapment | 2.9 (1.4) | 3.7 (1.1) | 10.5 (6.5) | 3 | 4.3 | 1.8 | 3 |
|  | Contact with exposed electrical connections | 2.9 (1.3) | 4.3 (0.9) | 12.7 (6.7) | 4 | 3.5 | 1.7 | 2 |
|  | *** Slips, trips and falls from heights | 3.5 (1.0) | 3.9 (0.9) | 13.9 (5.8) | 6 | 6.3 | 10.7 | 6 |
|  | *** Working on slippery surfaces | 4.0 (0.9) | 3.5 (0.9) | 14.0 (4.8) | 7 | 6.8 | 12.3 | 6 |
|  | Vehicle-related accident on the farm | 3.0 (1.2) | 4.2 (0.9) | 12.9 (6.2) | 5 | 3.2 | 3.0 | 1 |
|  | Exposure to noise | 3.3 (1.3) | 2.9 (1.1) | 10.3 (6.5) | 2 | 2.5 | NA | 0 |
|  | Working in poor lighting | 3.0 (1.1) | 2.8 (1.2) | 8.7 (5.6) | 1 | 1.7 | NA | 0 |
| environmental (h_env_ = 8) | *** Working in the sun | 4.1 (1.2) | 3.0 (1.0) | 12.7 (6.0) | 7 | 7.2 | 14.7 | 5 |
|  | *** Working in a cold environment | 3.8 (1.1) | 3.1 (0.9) | 12.0 (4.9) | 2 | 5.8 | 8.2 | 5 |
|  | *** Working in heavy rains | 3.7 (0.9) | 3.3 (1.0) | 12.4 (5.9) | 5 | 6.5 | 7.6 | 6 |
|  | Exposure to flooding | 3.2 (1.1) | 3.7 (1.0) | 12.4 (6.2) | 4 | 2.8 | NA | 0 |
|  | *** Exposure to landslides | 3.6 (1.1) | 4.2 (0.9) | 15.4 (6.3) | 8 | 5.2 | 9.6 | 4 |
|  | *** Exposure to storms / strong winds | 3.4 (1.1) | 3.6 (1.0) | 12.7 (6.5) | 6 | 3.5 | 6.0 | 1 |
|  | Exposure to a hailstorm | 3.4 (1.1) | 3.5 (0.9) | 12.3 (5.5) | 3 | 4.0 | 4.8 | 3 |
|  | Exposure to a forest fire | 2.4 (1.2) | 3.3 (1.2) | 7.7 (5.5) | 1 | 1.0 | NA | 0 |
| psychosocial (h_psy_ = 20) | *** Financial pressure from market | 4.2 (0.8) | 3.6 (0.9) | 15.1 (5.2) | 20 | 14.3 | 10.0 | 1 |
|  | Delayed payments | 3.7 (0.9) | 3.5 (0.7) | 13.3 (5.0) | 16 | 11.7 | NA | 0 |
|  | *** Unexpected events | 3.9 (0.9) | 3.4 (1.1) | 13.4 (6.0) | 17 | 16.2 | 14.8 | 3 |
|  | *** Limited access to farm inputs | 3.7 (0.8) | 3.3 (1.1) | 12.7 (5.5) | 12 | 17.0 | 11.2 | 4 |
|  | *** Failure of coffee crop | 3.8 (0.7) | 3.5 (1.0) | 13.2 (4.8) | 15 | 16.2 | 14.8 | 3 |
|  | *** Limited access to microcredits or loans | 3.9 (0.8) | 3.4 (1.0) | 13.6 (5.7) | 18 | 16.3 | 11.8 | 4 |
|  | *** Theft | 4.1 (0.7) | 3.6 (0.9) | 15.1 (5.0) | 19 | 15.8 | 9.3 | 3 |
|  | High certification requirements | 3.7 (0.8) | 3.4 (1.2) | 13.0 (6.4) | 13 | 8.7 | NA | 0 |
|  | High legal compliance requirements | 3.8 (1.0) | 3.3 (1.2) | 13.1 (6.9) | 14 | 8.7 | NA | 0 |
|  | Low availability of workforce | 3.4 (1.1) | 2.9 (1.0) | 10.6 (6.0) | 9 | 11.8 | NA | 0 |
|  | Social isolation / loneliness | 3.3 (1.1) | 2.8 (1.0) | 9.5 (5.5) | 5 | 4.7 | NA | 0 |
|  | Not enough resting breaks | 3.5 (1.0) | 3.1 (0.8) | 11.5 (5.7) | 10 | 13.8 | 6.0 | 3 |
|  | Fast-paced work | 3.4 (1.0) | 2.8 (1.1) | 9.8 (5.7) | 7 | 10.0 | 2.5 | 1 |
|  | Unpredictable working hours | 3.6 (0.7) | 3.1 (1.1) | 11.7 (6.2) | 11 | 13.7 | 5.3 | 2 |
|  | Conflict with family members | 3.2 (1.1) | 2.9 (1.0) | 10.0 (6.4) | 8 | 9.8 | NA | 0 |
|  | Conflicts with co-workers | 3.1 (1.0) | 2.8 (1.0) | 9.4 (6.0) | 3 | 6.5 | NA | 0 |
|  | Workplace violence | 3.1 (1.0) | 2.9 (1.1) | 9.6 (6.2) | 6 | 5.0 | NA | 0 |
|  | Bullying | 2.6 (1.2) | 2.5 (1.1) | 7.7 (6.8) | 1 | 1.8 | NA | 0 |
|  | Sexual harassment/assault | 2.7 (1.1) | 3.4 (1.1) | 9.5 (5.9) | 4 | 4.0 | NA | 0 |
|  | Social discrimination | 2.9 (1.2) | 2.9 (1.0) | 9.0 (6.2) | 2 | 4.0 | NA | 0 |
